# The preventive effect and mechanism of Tibetan medicine *Aconitum tanguticum* (Maxim.) Stapf on acute lung injury

**DOI:** 10.1186/s13020-025-01072-7

**Published:** 2025-02-12

**Authors:** Xiang Meng, Yu-Peng Liu, Jia-Wei Dai, Yuan Bai, Xin Hu, Muhammad Azhar, Xian-Ju Huang

**Affiliations:** 1 College of Pharmaceutical Science, South-Central Minzu University, 182 Minyuan Road, Wuhan, 430074 People’s Republic of China; 2 Department of Genomics and Bioinformatics, Cholistan University of Vertienary Sciences Bahwalpur, 63221 Bahwalpur, Pakistan; 3 Hubei International Science and Technology Cooperation Base (SH2311), Wuhan, 430074 China

**Keywords:** *Aconitum tanguticum* (Maxim.) Stapf, Acute lung injury, Inflammation, Ferroptosis

## Abstract

**Ethnopharmacological relevance:**

*Aconitum tanguticum* (Maxim.) Stapf (ATS) is a rare Tibetan medicinal plant that belongs to the Ranunculaceae family. This herb is mainly distributed in the high-altitude areas of Qinghai, Gansu provinces, and Tibetan Autonomous Region in China. In Tibetan medicine, ATS is mainly used to treat lung inflammation, hepatitis, gastrointestinal diseases, influenza, fever caused by infectious diseases, food poisoning, snake and scorpion bites, and yellow water disease. ATS has anti-inflammatory, antiviral, and other pharmacological effects, according to recent research. It is welltolerated by individuals from diverse ethnic groups and has a long history of use in Tibetan medicine.

**Aim of the study:**

This study investigated the preventive effects of ATS alcoholic extract on acute lung injury (ALI) in mice and aimed to elucidate its possible mechanism of action.

**Materials and methods:**

Alveolar epithelial cells A549 and specific pathogen-free C57BL/6 mice were induced with lipopolysaccharide (LPS) to establish ALI models both in vivo and in vitro and to explore the pharmacological effects and therapeutic mechanisms of ATS.

**Results:**

ATS down-regulated the mRNA levels of inflammatory factors NF-κB p65, TNF-α, IL-1β, and IL-8, inhibited the release of reactive oxygen species, inhibited epithelial-mesenchymal transition caused by sustained cell injury, promoted the Keap1/Nrf2/HO-1 signalling pathway, reduced the degree of oxidative stress in vivo, and inhibited the production of proteins associated with LPS-induced ferroptosis.

**Conclusion:**

The Tibetan medicine ATS reduced pulmonary haemorrhage and oedema in ALI mice, alleviated the degree of lung tissue lesions, inhibited the expression of inflammatory factors and apoptosis, and plays a preventive role against acute lung injury in mice.

**Graphical Abstract:**

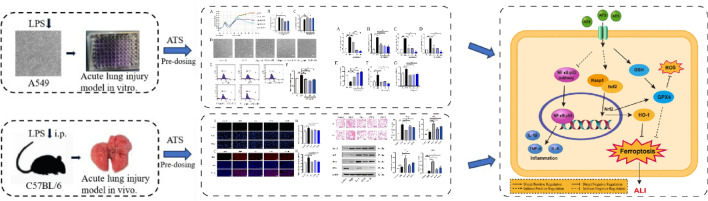

## Introduction

Severe cases of the coronavirus disease (COVID-19) develop into severe illness, often manifesting as acute lung injury (ALI). Epithelial and capillary cells in the bronchi rupture, forming pulmonary oedema and fibrosis, leading to severe respiratory insufficiency, becoming typical clinical features of patients with severe COVID-19 [1], which is a relatively serious form of disease. The prognosis of patients with COVID-19 is directly affected by the onset and development of ALI. Clinical disease mortality in ALI is relatively high as it involves extensive inflammatory damage and pulmonary dysfunctions with a global mortality rate as high as 50% [2]. Clinical interventions for the treatment include negative fluid regulation [3], mechanical ventilation [4], glucocorticoids [5], nitric oxide ventilation [6], and antibiotics [7]. However, all of these methods can have a notable impact on a patient’s quality of life. Therefore, there is a need to find an effective treatment for ALI that has less impact on the patients’ quality of life.

The main features of alveolar epithelial cells in ALI are the formation of a persistent inflammatory responses, release of numerous chemokines and pro-inflammatory cytokines, and induction of subsequent pathological processes such as pulmonary fibrosis [8]. The Keap1/Nrf2/HO-1 pathway is one of the most critical endogenous antioxidative stress pathways and an important target for inflammation-related diseases [9]. Simultaneously, some studies have reported that ferroptosis plays an important role in the progression of ALI and inflammatory injury [10].

*Aconitum tanguticum* (Maxim.) Stapf (ATS), a rare medicinal plant in Tibetan medicine, belongs to the genus *Aconitum*, family Ranunculaceae. In the early days of the COVID-19 epidemic, Tibetan Autonomous Region, Qinghai province, and other areas that inherited Tibetan medicine included traditional Tibetan medicines, such as Twelve Flavours Yishou Powder, Twenty-Five Flavours Lung Disease Capsules, Flu Pills, and Twenty-Five Flavours Main Medicine Powder, in their prevention and control plans. ATS prescription has a good preventive effect on COVID-19, particularly in preventing the “inflammatory storm”. This outcome resulted in the clinical application of Tibetan medicine in COVID-19 treatment [11] and increased an interest in ATS research. The ALI mouse model induced by lipopolysaccharide (LPS) is consistent with the pathological manifestations of COVID-19. In this study, an LPS-induced ALI mouse model was used to investigate the anti-inflammatory effect of ATS in the treatment of ALI. Specifically, the study aimed to determine whether ATS exerts its anti-inflammatory role by regulating the Keap1/Nrf2/HO-1 pathway and inhibiting ferroptosis.

## Materials and methods

### ATS preparation

Dr. Qien Li of Qinghai University donated and helped with the identification of ATS samples (specimen no. LQE-2019-066). A 150 g sample of dried ATS was obtained, crushed, passed through a no. 30 sieve, and then immersed in ethanol (0.97 volume fraction) at a ratio of five times the volume at room temperature (26 ℃) for 24 h. This process was repeated six times followed by filtration to remove the contaminants. The solvent was evaporated using a rotary evaporator (Great Wall Science, Industry & Trade Co. Zhengzhou, China) and 11.73 g of ATS extract were obtained.

### Liquid chromatography-tandem mass spectrometry (LC–MS/MS) identification

Methanol (80%) was used as the solvent to dissolve an appropriate amount of ATS; the sample was ultrasonicated for 30 min (250 W, 40 kHz) until complete dissolution. The sample was then transferred to a 50 mL volumetric flask, and the final volume was made up to 50 mL, cooled to room temperature, and then filtered using a 0.22 μm microporous membrane to obtain the test solution.

LC-MS/MS identification was performed using a Thermo Fisher UltiMate 3000 high performance liquid chromatography instrument (HPLC). An Accucore aQ 100 × 2.1 mm 2.6 µm HPLC column was used. The mobile phase consisted of two phases: A and B. A was acetonitrile and B was 0.1% aqueous formic acid with the following gradient elution program: 0–3 min, 5–10% A; 3–7 min, 10–17% A; 7–9 min,17–20% A; 9–11 min, 20–24% A; 11–14 min, 24–28% A; 14–16 min, 28–30% A; 16–19 min, 30–35% A; 19–21 min, 35–37% A; 21–29 min, 37–55% A; 29–33 min, 55–63% A; 33–43 min, 63–95% A; 43.1–50 min, 95–5% A. The flow rate was 0.2 mL/min and the column temperature was 25 °C. For MS, the ion source was a heated electrospray ion source with positive and negative ion detection modes. The positive and negative spray voltages were 3200 V each. Capillary temperature was 300.00 °C, gas volume flow was 40.00 Arb, and auxiliary gas volume flow was 10.00 Arb. The maximum injection current was 100.00 μA, the probe heater temperature was 320.00 °C, and the scanning range m/z was 80–1200. Finally, the Xcalibur software (version 4.0) was used to calculate the high-resolution accurate mass number, fit the molecular formula, and match it to the mzCloud database.

### Evaluation of medication efficacy and cell culture

The alveolar epithelial cell line A549 was obtained from the Wuhan University Cell Bank. Cells were cultured in an incubator (Heal Force, Hong Kong) at 37 °C in Dulbecco’s Modified Eagle Medium (DMEM) high-sugar culture medium (8122509; Gibco, CN) supplemented with 10% foetal bovine serum (FBS) (22100702; Zhejiang Tianhang Viotechnology Co., Lod, CN). A549 cells were seeded in 96-well plates at a density of 5 × 10^3^ cells/well and cultured until they reached logarithmic growth phase. Subsequently, cells were exposed to LPS (22039849; Sigma-Aldrich, USA) at 1–100 μg/mL for 24 h. Following this treatment, the culture medium was replaced with DMEM supplemented with 2% FBS. To establish the final mould-making concentration, cell viability was measured using a real-time label-free cell analyser (Agilent Technologies, USA). To determine the range of supplied concentrations, the cells were treated with final concentrations of 1–100 μg/mL ATS for 12 h under standard medium conditions. Cell viability was assessed using the MTT assay (8122509; Biosharp, CN).

To evaluate LPS-induced cell proliferation, A549 cells were seeded on E16 plates (ACEA, USA) at a density of 5 × 10^3^ cells/well. The xCellingence RTCA S16 system analysed cell proliferation in real time and the cell index was expressed by monitoring changes in the impedance of the electrode interface in real time.

In the control, model (LPS 100 μg/mL), and medication administration groups, A549 cells were injected into 96-well plates at a rate of 100 μL/well. ATS (5–20 μg/mL) pretreatment was administered to the medication group for 12 h. LPS (100 μg/mL) was added to the medium and cultured for 24 h before being replaced with DMEM supplemented with 2% FBS. The growth rate of the cells was assessed using the MTT assay.

### Monitoring cell morphology and ROS detection

A549 cells were seeded in 6-well plates and treated as described in the previous section. Following delivery, each batch of cells was rinsed with PBS to remove excess liquid, and the cellular morphology was assessed using an inverted microscope (OPTEC, Chongqing, China).

A549 cells were seeded in 6-well plates and treated with medication, and ROS levels were measured using a ROS detection kit (S0033S; Beyotime, CN). The cells were immediately treated with 10 μM DCFH-DA and incubated for 30 min. Intracellular ROS levels were determined using flow cytometry by analysing the relative mean fluorescence intensity values of each set of cells.

### Animal treatment

Liaoning Changsheng Biotechnology Co. provided specific pathogen-free (SPF) C57BL/6 mice (weight, 20 ± 2 g; 60 males; experimental animal licence number SCXK(Liao)2020-0001). The mice were housed in SPF-grade animal barriers at the Experimental Animal Center of the South-Central Minzu University. All procedures involving the use of experimental animals were carried out in strict compliance with the rules established by the Animal Ethics Committee of South-Central Minzu University and international National Institute of Health standards. The ATS dosage was determined according to the relevant documents [12]. To obtain a medication suspension, 0.5% sodium carboxymethyl cellulose (CMC-Na; 20150421; Sinopharm Chemical Reagent Co., Ltd., CN) was added. Before the experiment began, all animals were acclimatized for seven days, and then randomly assigned into five groups (12 mice in each group).

### Drug administration and collection of lung tissue samples

The trial involved pre-dosing throughout the course of a 14-day dosing cycle. The control and model groups were orally administered a 0.5% CMC-Na solution, and the ATS group was orally administered the corresponding concentration of ATS for 14 consecutive days (low-dose group: 350 mg/kg of ATS, hight-dose: 700 mg/kg of ATS). On day 12 of the experiment, the ferroptosis inhibitor group received an intraperitoneal injection of Fer-1 (5 mg/kg, RM02804, ABclonal Technology, CN), which acted for 24 h over the course of three days. Six hours after the last dose of the drug, LPS (10 mg/kg) was injected to all groups of mice except the normal group for a 12-h modelling period [13].

Upon completion of the modelling process, the mice were sacrificed, and the left lung lobe was excised to determine the wet/dry weight ratio (W/D) of the mouse lung tissue. To facilitate histological examination, a portion of the right lung lobe was preserved in a 4% paraformaldehyde solution (20190428; Sinopharm Chemical Reagent Co., Ltd., CN) at room temperature. The remaining tissue was stored in an ultra-low-temperature refrigerator (Haier, China) at − 80 °C for further studies.

### Wet to dry weight ratio of mouse lung tissue (W/D)

After the mice were sacrificed, fresh lung tissue was removed and any unwanted tissue remnants were removed and rinsed in saline. The wet weight of the left lung tissue was determined after removing excess water using a filter paper. Subsequently, the tissue was subjected to a drying process at a constant temperature of 60 °C for 48 h in a specialised oven (Ruihua Instrument Equipment Co., Wuhan, China) for dry weight determination. W/D was determined for each mouse lung using the data collected.

### Histopathological staining of lung tissue

Fresh lung tissue was soaked for over 24 h in 4% paraformaldehyde. To create 5 μm paraffin sections, the tissue was excised and paraffin embedded. Haematoxylin and eosin (H&E) staining was used to stain the sections. Subsequently, the staining of each segment was meticulously examined under a light microscope, enabling comparative analysis of the pathological alterations in the lung tissue across different groups of mice.

### Myeloperoxidase (MPO) activity in mouse lung tissue

Mouse lung tissue was collected and rapidly ground at a low temperature, and MPO activity was determined using a colorimetric MPO test kit according to the manufacturer’s instructions (A044-1-1; Nanjing Jiancheng Institute of Biological Engineering Co., Ltd., CN).

### ROS fluorescence labelling and TUNEL

Xylene was used to soak the tissue slices at room temperature. The sections were then dehydrated using an ethanol gradient and washed with PBS. Histochemical pens were used to draw circles on the edges of tissues to facilitate further manipulation. After adding PBS to wash the sections, the membrane-breaking working solution was added and incubated for 20 min before adding the proteinase K working solution at room temperature. Buffer was added drop-wise and allowed to equilibrate for 10 min. The recombinant TDTase, dUTP, and buffer were combined in a reaction solution at 1:5:50 L (v/v/v). The resulting mixture was subsequently incubated at a temperature of 37 °C for 2 h. After staining with DAPI for 10 min at room temperature, the sections were sealed. Subsequently, the sections were examined under a fluorescence microscope and images were captured using a DAPI UV excitation wavelength of 330–380 nm, CY3 excitation wavelength of 510–561 nm, and red light at 590 nm.

Frozen mouse lung tissue samples were prepared, rehydrated at room temperature, and dried. A bursting agent was applied to the circle created using a histochemical pen, which was then allowed to burst for 5 min and cleaned. ROS staining solution was added drop-wise and incubated at constant temperature (37 °C) in the dark for 30 min. Fluorescence microscopy was used to capture images at specific excitation and emission wavelengths for each fluorophore (DAPI: excitation 330–380 nm, emission 420 nm; FITC: excitation 465–495 nm, emission 515–555 nm; CY3: excitation 510–560 nm, emission 590 nm). Prior to imaging, the nuclei were re-stained with DAPI, shaken to remove excess moisture, and sealed using an antifluorescent bursting agent.

### Measurement of tissue iron content and oxidative stress indicators

The remaining frozen lung was used to extract lung tissue. The supernatant of the lung tissue homogenate was obtained after mixing in a high-speed, low-temperature tissue grinder with saline at a specific ratio. SOD (A001-3-2), GSH (A006-2-1), CAT (A007-1-1), and tissue iron levels were assessed according to the kit’s manufacturer’s instructions (Nanjing Jiancheng Institute of Biological Engineering Co., Ltd., CN).

### Quantitative real-time PCR (qRT-PCR)

The mRNA expression levels of inflammation- and epithelial-mesenchymal transition (EMT)-associated genes in A549 cells were determined using qRT-PCR, with GAPDH as the reference gene. Total RNA was extracted from cell samples using TRIzol reagent (W9712; TIANGEN, CN), and cDNA was synthesised by reverse transcription using a kit (962103G2626; ABclonal Technology, CN). The qRT-PCR (AL13557A; TaKaRa, JPN) conditions were as follows: denaturation at 95 °C for 3 min, followed by 40 cycles at 95 °C for 5 s and 60 °C for 34 s.

The relative expression levels of TNF-, IL-1β, and IL-6 mRNA in the lung tissues of each group of mice were similarly determined using 20–30 mg of tissue. The primer sequences used are listed in Table 1.

**Table 1:** Primer sequences for the target genes used in the experiment

	Primer 5′-3′	T
Gene (for A549)		
* GAPDH*	F: GCACCGTCAAGGCTGAGAAC R: GACTCCACGACATACTCAGCAC	61 ℃
* TNF-α*	F: CCTCTCTCTAATCAGCCCTCTG R: GAGGACCTGGGAGTAGATGAG	58 ℃
* IL-1β*	F: ATGATGGCTTATTACAGTGGCAA R: GTCGGAGATTCGTAGCTGGA	57 ℃
* IL-8*	F: ACTGAGAGTGATTGAGAGTGGAC R: AACCCTCTGCACCCAGTTTTC	60 ℃
* E-cadherin*	F: CGAGAGCTACACGTTCACGG R: GGGTGTCGAGGGAAAAATAGG	60 ℃
* MUC5AC*	F: CAGCACAACCCCTGTTTCAAA R: GCGCACAGAGGATGACAGT	59 ℃
* α-SMA*	F: TGTGGCTATCCAGGCGGTGC R: TCTCGGCCAGCCAGATCCAGAC	65 ℃
Gene (for mice)		
* GAPDH*	F: ACCCAGAAGACTGTGGATGG R: TCAGCTCAGGGATGACCTTG	57 ℃
* TNF-α*	F: TTGACCTCAGCGCTGAGTTG R: CCTGTAGCCCACGTCGTAGC	59 ℃
* IL-1β*	F: CAGGATGAGGACATGAGCACC R: CTCTGCAGACTCAAACTCCAC	58 ℃
* IL-6*	F: ACTCACCTCTTCAGAACGAATTG R: CCATCTTTGGAAGGTTCAGGTTG	58 ℃

### Western blot analysis

Lung tissue was removed on ice. Mouse lung tissues were homogenised and treated for 30 min with RIPA buffer containing a protease inhibitor cocktail. Total protein was extracted using differential centrifugation (13,000 *g*, 15 min at 4 °C) and quantified using a BCA assay kit (CW0014S, Cowin Biotech Co., Ltd., CN). To denature the protein samples, 5 × SDS-PAGE protein upload buffer was added proportionally to the samples (100 ℃, 5 min). For electrophoresis (SDS-PAGE), identical amounts of total denatured protein (40 µg) were added to each group. After electrophoresis and transfer, the immunoreactive strip was blocked with 5% (w/v) skimmed milk. The strips were incubated with anti-Keap1, anti-Nrf2, anti-HO-1, and anti-GPX4 antibodies (1:1000) overnight at 4 °C, and then incubated with secondary antibodies (HRP conjugated Goat Anti-Mouse IgG, GB23301, Servicebio, CN) at room temperature for 30 min. The signal was detected in the immunoreactive strips using enhanced chemiluminescence with an ECL test kit (ABP Biosciences, USA). ImageJ was used for quantification using grayscale images.

### Statistical analysis

Statistical analysis was performed using the SPSS software (version 18.0). Data are presented as mean ± standard deviation. Multiple comparisons were performed using a one-way analysis of variance and Dunnett’s test. Statistical significance was set at *P* < 0.05.

## Results

### LC–MS/MS identification results

**Fig. 1:**
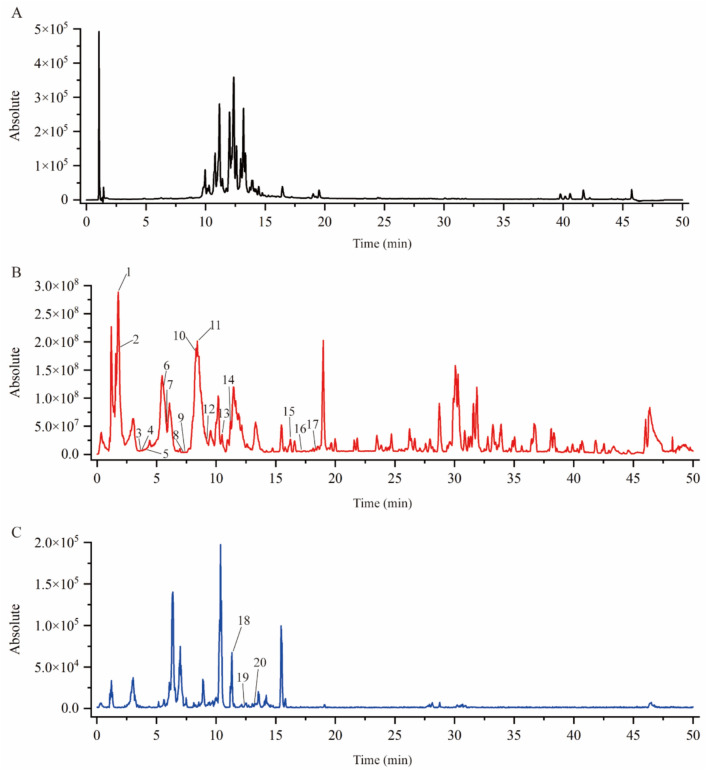
Total ion chromatogram, positive ion mode and negative ion mode of LC–MS/MS detection: **A** total ion chromatogram; **B** positive ion mode; **C** negative ion mode

To determine the chemical composition of ATS, LC-MS/MS was used to identify its components. Primary and secondary mass spectrometry data were compared with the mzCloud database and the literature. A total of 20 compounds were identified, including 17 in positive ion mode and 3 in negative ion mode. The LC-MS/MS identification map is shown in Fig. 1, and compound information is presented in Table 2.

**Table 2:** Chemical composition of ATS

No.	Identification	Molecular formula	*t*_R_ (min)	Ion mode	Theoretical value	Measured value	Error (× 10^–6^)	Fragment ion
1	n-Methyltyramine	C9H13NO	1.78	[M + H]^+^	152.107	152.107	0.004	121.06, 103.05
2	9-hydroxynominine/tongolinine	C20H27NO2	1.81	[M + H]^+^	314.211	314.211	2.341	296.15, 121.07
3	Tangutisine	C20H27NO4	3.56	[M + H]^+^	346.201	346.201	2.180	310.18, 247.15
4	Hetisinone	C20H25NO3	3.76	[M + H]^+^	328.191	328.190	2.286	310.18
5	Tyramine	C8H11NO	4.01	[M + H]^+^	138.091	138.092	−3.906	122.07, 121.08
6	Heteratisine	C22H33NO5	5.54	[M + H]^+^	392.243	392.242	2.018	360.22, 342.21
7	Hordenine	C10H15NO	5.84	[M + H]^+^	166.123	166.122	2.713	147.047, 121.06, 119.05, 91.05
8	Heterophyllidine	C21H31NO5	7.35	[M + H]^+^	378.227	378.227	2.061	360.25
9	Tanguticuline E	C20H25NO4	7.46	[M + H]^+^	344.186	344.185	1.409	326.21, 121.07
10	Tanwusine	C24H35NO6	8.14	[M + H]^+^	434.254	434.253	2.359	312.20, 294.19
11	6-benzoylheteratisine	C22H33NO2	8.42	[M + H]^+^	344.258	344.258	1.905	342.21, 307.23
12	Atisine	C20H27NO3	9.17	[M + H]^+^	330.206	330.206	2.151	326.25
13	13-o-acetylhetisine/11-acetylhetisine	C22H29NO4	10.56	[M + H]^+^	372.217	372.216	1.759	312.20
14	Acorine	C22H29NO5	10.64	[M + H]^+^	388.212	388.212	− 0.079	328.19, 310.18
15	Navirine B	C30H40N2O3	16.98	[M + H]^+^	477.311	477.311	0.355	459.30, 121.06
16	Heterophylline	C21H31NO4	17.04	[M + H]^+^	362.233	362.232	2.719	344.19
17	Phenylalanine	C9H11NO2	18.30	[M + H]^+^	166.086	166.086	2.139	149.13, 121.07
18	Quercetin-3-o-[β-d-glucose-(1 → 3)-(4-o-trans-p-coumaroyl)]-α-l-rhamnose-(1 → 6)-β-d-mannose-7-o-β-d-grape Glucosides	C45H60O30	11.33	[M−H]^−^	1079.310	1079.309	1.013	917.25, 771.18, 609.15, 301.03
19	Quercetin-3-o-[β-d-glucose-(1 → 3)-(4-o-E-p-coumaroyl)]-α-l-rhamnose-(1 → 6)-β-d-galactoside-7-o-α-l-rhamnoside	C48H56O27	12.36	[M−H]^−^	1063.294	1063.293	0.385	609.14, 301.03
20	Kaempferol-3-o-[β-d-glucose-(1 → 3)-(4-o-E-p-coumaroyl)]-α-l-rhamnose-(1 → 6)-β-d-glucose-7-o-α-l-rhamnose Glycosides	C48H56O26	13.22	[M−H]^−^	1047.299	1047.298	0.234	593.15, 285.04

### ATS inhibits LPS-induced A549 cell injury and the release of intracellular ROS

**Fig. 2:**
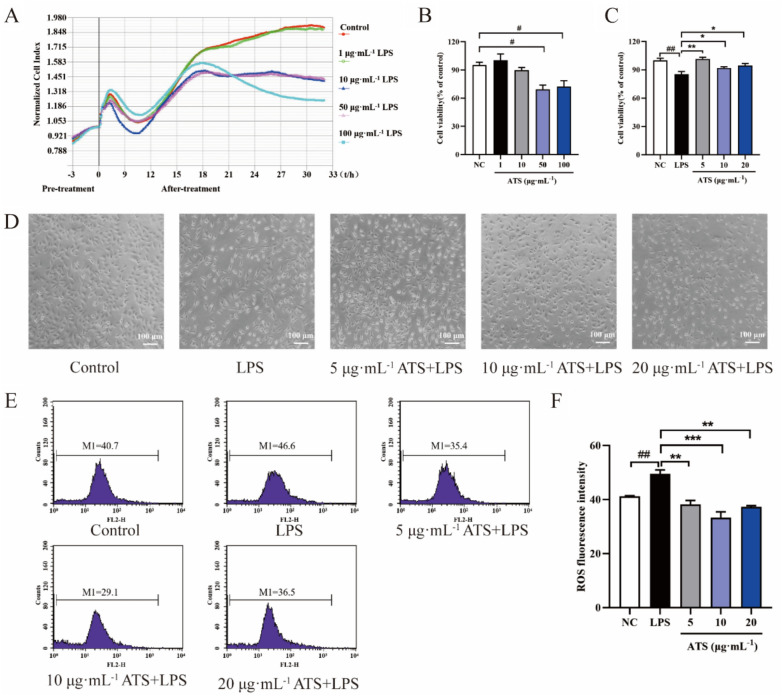
**A** RTCA analysis of A549 cell proliferation on E-16 plate; **B** analysis of A549 cell proliferation; **C** effect of ATS on A549 cell viability induced by LPS; **D** change of cell morphology. n = 6; ^#^*P* < 0.05, ^##^*P* < 0.01 compared with normal control group; *P < 0.05, **P < 0.01 compared with LPS group. **E** flow cytometry analysis of LPS-induced changes in intracellular ROS levels. **F** relative fluorescence intensity analysis; n = 3; ^##^*P* < 0.01, compared with normal control group; **P < 0.01, ***P < 0.001 compared with LPS group

Detection of the effect of different concentrations of LPS on the viability of A549 cells using real-time unlabelled cell analysers showed that LPS was the most significant suppressor of cell viability at 100 μg/mL (Fig. 2A), so it was the optimal modelling condition for this experiment. Drug safety and effect of ATS on cell viability was determined using the MTT method, and the results revealed that ATS did not show significant toxicity below 50 μg/mL (Fig. 2B); MTT results revealed that ATS administration could reduce the damage of LPS to A549 cells, as shown in Fig. 2C. The LPS-treated group produced a more stretched or elongated fusiform morphology and an increased number of dead cells compared to the control group, and ATS was able to reverse this phenomenon (Fig. 2D).

After treatment with different concentrations of ATS on A549 cells for 12 h, they were stimulated with LPS for 24 h, and intracellular ROS levels were measured after the cells were collected. The results are shown in Fig. 2E–F. Compared with the normal control group, LPS significantly up-regulated the intracellular ROS level (*P* < 0.01), whereas pretreatment with different ATS concentrations (5, 10, and 20 μg/mL) inhibited ROS levels in LPS-induced A549 cells (*P* < 0.01, *P* < 0.001). The ROS level in the ATS (10 μg/mL) group decreased more significantly than that in the ATS (20 μg/mL) group.

### ATS inhibits LPS-induced expression of NF-κB p65, TNF-α, IL-1β, and IL-8 and EMT-related factor mRNA expression induced by persistent inflammation

**Fig. 3:**
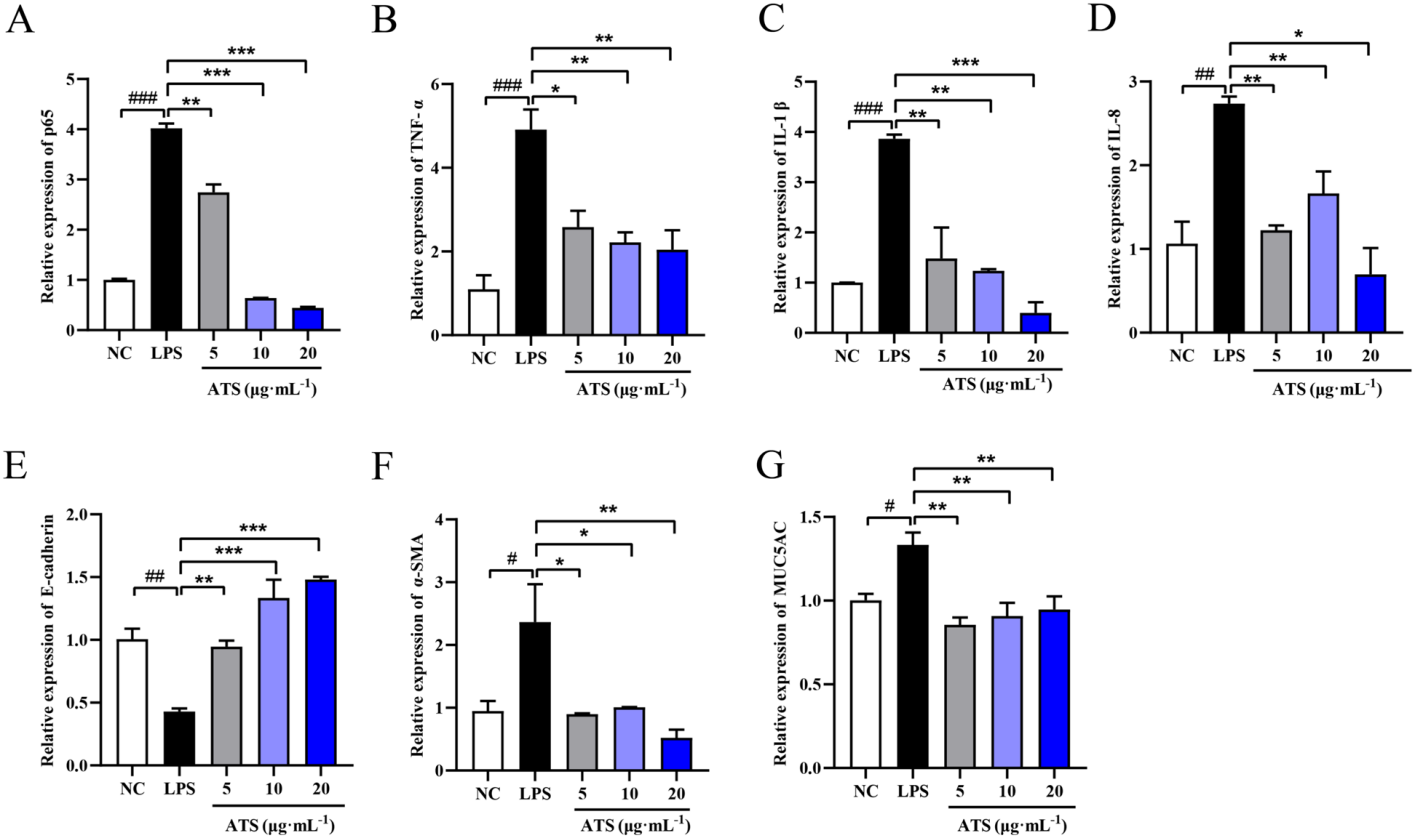
**A**–**G** shows the mRNA expressions of the following genes in cells: NF-κB p65, TNF-α, IL-1, IL-8, E-cadherin, α-SMA, and MUC5AC, respectively. The normal control group (NC) and the model group (LPS) are represented by the letters, respectively. n = 5; ^##^*P* < 0.01, ^###^*P* < 0.001 compared with normal control group; *P < 0.05, **P < 0.01, ***P < 0.001 compared with LPS compared to the model group

As shown in Fig. 3A–D, LPS induced inflammatory injury in A549 cells, and intracellular NF-κB p65, TNF-α, IL-1β, and IL-8 mRNAs levels were significantly up-regulated (*P* < 0.001, *P* < 0.01). The expression of inflammatory factors in ATS-pretreated A549 cells was significantly reduced in a dose-dependent manner (*P* < 0.001, *P* < 0.01, *P* < 0.05). In this experiment, the EMT indicators were also detected after sustained inflammatory injury of the cells, as shown in Fig. 3E–G. After continuous stimulation of A549 cells with LPS for 24 h, the intracellular E-cadherin mRNA level was inhibited (*P* < 0.01), and the α-smooth muscle actin and mucin mRNA levels increased (*P* < 0.05), indicating that persistent inflammation may induce the EMT in A549 cells, and the above phenomenon was reversed after ATS intervention.

### Impact of ATS on the pathomorphology of mice with LPS-induced ALI

**Fig. 4:**
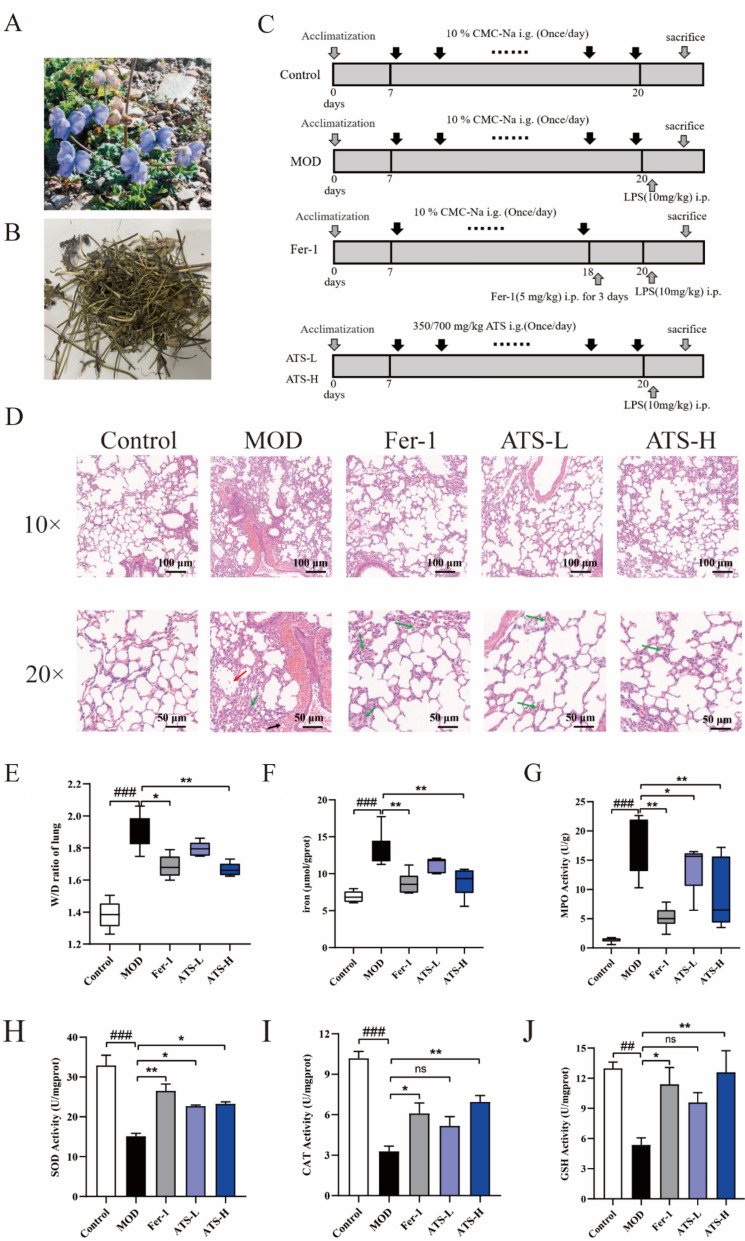
**A**, **B** The plant of Tibetan medicine ATS, **C**: The schematic diagram of animal treatment, **D**–**J**: Effect of ATS on pathomorphology; W/D ratio; iron content; MPO activity; activity of SOD, CAT, GSH content in the lung tissue; n = 6; compared with normal control group, ^##^*P* < 0.01; compared with model group, **P < 0.01, *P < 0.05

Figure 4C depicts the animal modelling procedure (for further information, refer to the Materials and methods section). Figure 4D displays the results of H&E staining of lung tissue. The lung tissue in the model group showed clear signs of inflammatory cell infiltration (red arrow), alveolar congestion (black arrow), collapse of the alveolar cavity, and other pathological events compared with the normal control group. The alveolar spacing was significantly widened, alveolar wall thickness was increased, and lung tissue structure was disturbed (green arrow). Compared with the model group, the pathology of lung tissue in the low- and high-dose groups was relieved to different degrees, inflammatory cell infiltration was relieved, and some alveolar structures remained intact, which was consistent with the effect of the Fer-1 group.

As shown in Fig. 4E–G, compared with the normal control group, the W/D ratio, tissue iron ion content, and MPO activity were significantly increased in the model group (*P* < *0*.001), which was consistent with the effect observed in the Fer-1 group. ATS reduced the W/D ratio, tissue iron content (*P* < *0*.01), and MPO activity in a dose-dependent manner. The results showed that ATS improved lung oedema in ALI mice and reduced the levels of Fe^2+^, a marker of iron-induced cell death. In addition, ATS inhibited the activation and migration of neutrophils in the lung tissues of mice, thereby attenuating the inflammatory response in the lungs.

SOD, CAT, and GSH activities in the lung tissues of mice in each group are shown in Fig. 4H–J. Compared with the normal control group, SOD, CAT, and GSH activities were significantly decreased in the model group (*P* < 0.01). ATS can reverse this phenomenon, thereby strengthening the tissue and cellular tolerance to oxidative stress.

### Effect of ATS on LPS-induced apoptosis and ROS generation in the lung tissue of ALI mice

**Fig. 5:**
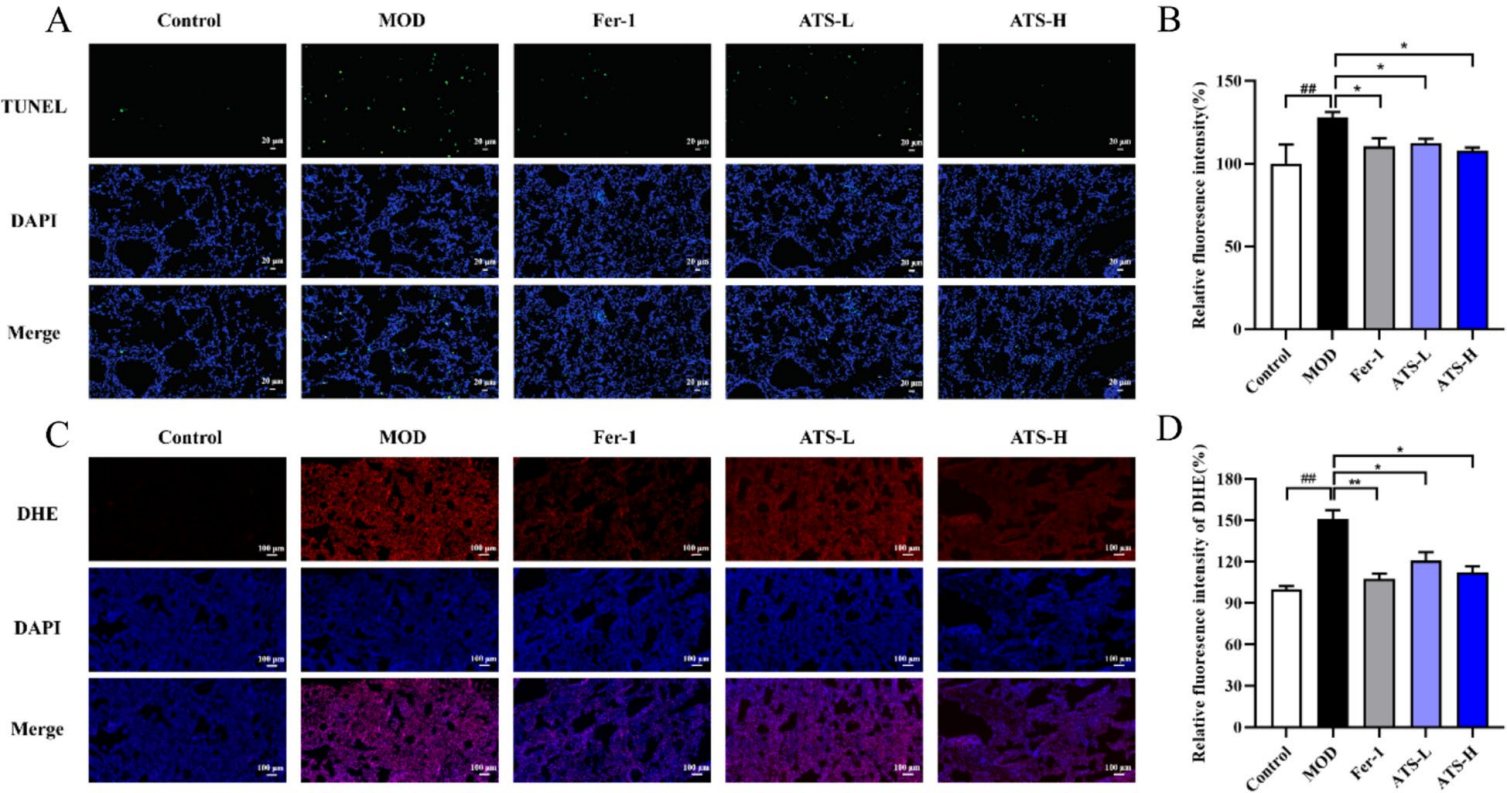
**A** Effect of ATS on apoptosis in lung tissue of ALI mice induced by LPS; **B** quantification results of relative fluorescence intensity; **C** effect of ATS on ROS generation in lung tissue of ALI mice induced by LPS **D** relative fluorescence Intensity quantification results; n = 3; compared with normal control group, ^#^*P* < 0.05; compared with model group, *P < 0.05

Apoptosis is an important pathological feature in ALI. To investigate the effect of ATS on LPS-induced ALI mouse lung tissue, TUNEL staining was used to detect the apoptosis in the lung tissue, as shown in Fig. 5A and B. The number of TUNEL-positive cells (green fluorescence) in the model group increased significantly compared with that in the normal control group, indicating that the cells in the lung tissues of mice with lung injury were apoptotic. After ATS intervention, the number of apoptotic cells in the lung tissue of ALI mice was significantly lower than that in the model group, which was consistent with the effect of the Fer-1 group.

A DHE fluorescent probe was used in the experiment. When cells were treated with DHE, they reacted with oxygen free radicals and emitted red fluorescence at a specific excitation wavelength. As shown in Fig. 5C and D, oxygen free radicals in the model group were significantly increased, whereas the fluorescence of oxygen free radicals in the normal control group was hardly observed. Compared with the model group, low-dose ATS significantly reduced the level of oxygen free radicals in the lung tissue of ALI mice, and the effect of high-dose ATS was more significant. The inhibitory effect of ATS on oxygen free radical levels in the lung tissues of ALI mice was consistent with that observed in the Fer-1 group.

### Regulatory effect of ATS on Keap1/Nrf2/GPX4/HO-1 protein expression and inflammatory factors in lung tissue of LPS-induced ALI mice

**Fig. 6:**
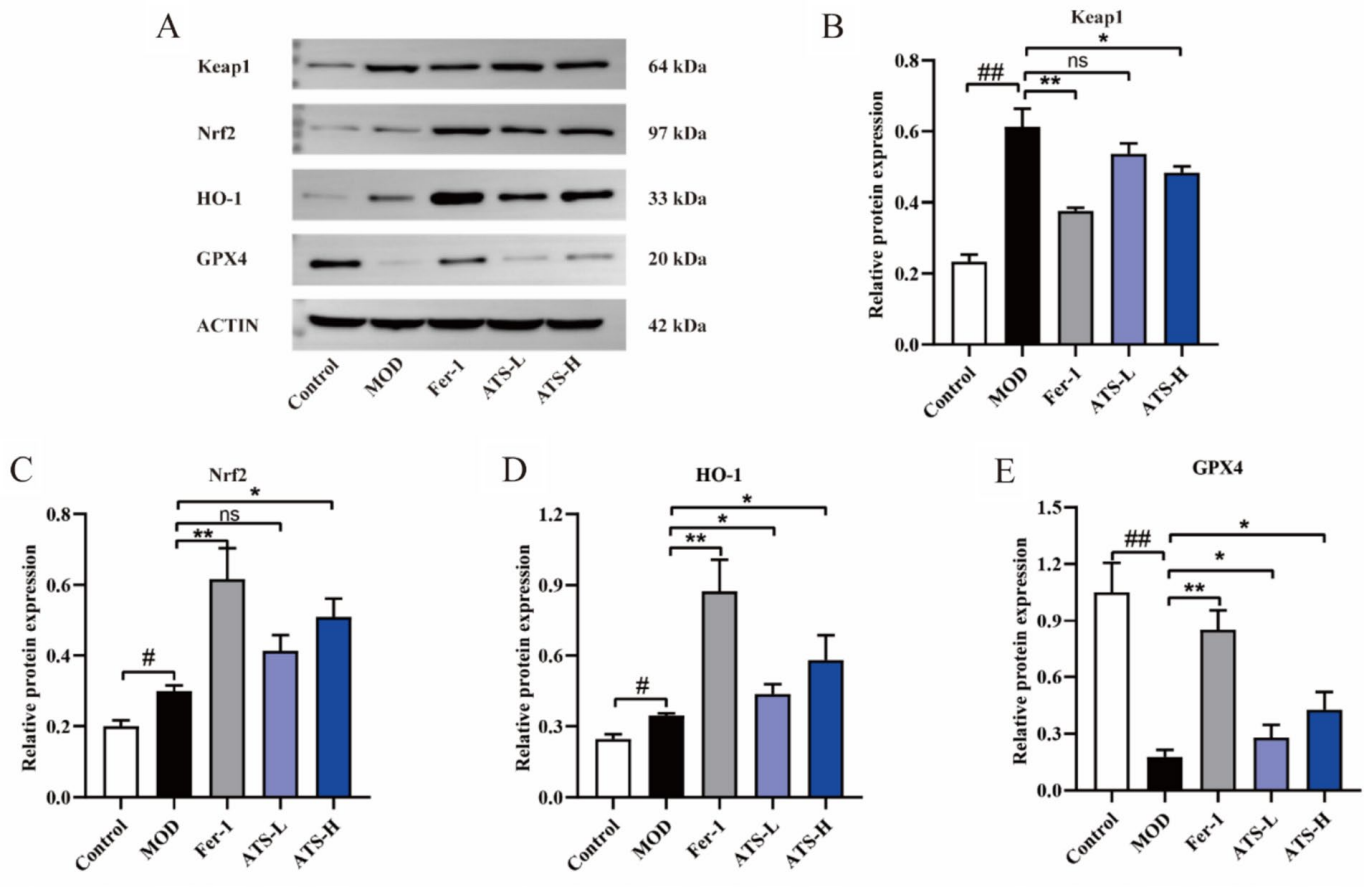
The effect of ATS on the mRNA expression of inflammatory factors and the protein level of Keap1/Nrf2/GPX4/HO-1 in the lung tissue of ALI mice induced by LPS: **A** Western blotting of Keap1/Nrf2/GPX4/HO-1; **B**–**E** Protein expression levels of Keap1, Nrf2, HO-1 and GPX4; n = 3; compared with normal control group, ^##^*P* < 0.01, ^#^*P* < 0.05; compared with model group, **P < 0.01, *P < 0.05

**Fig. 7:**
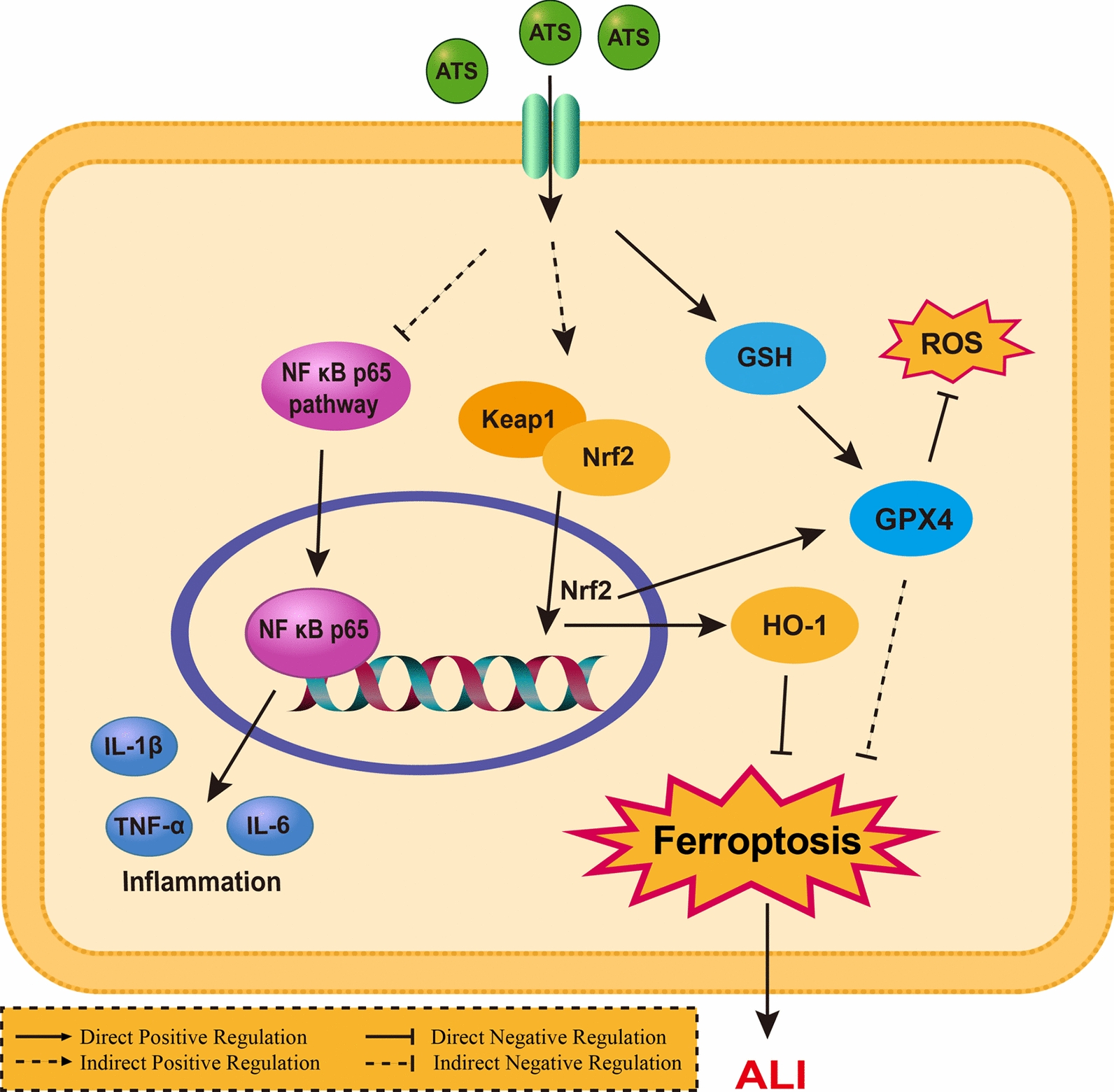
Preliminary speculation on the signaling pathways involved in the mechanism of ATS on ALI

In lung tissue, the Keap1/Nrf2/GPX4/HO-1 protein enhances tissue antioxidant capacity and protects tissue cells from oxidative stress injury [14]. GPX4 and Nrf2 negatively regulate iron-related cell death by reducing ROS production and inhibiting intracellular iron uptake, respectively [15]. ATS plays an important role in LPS-induced ALI in mice. As shown in Fig. 6A–E, compared to the normal control group, the protein expression of Keap1 in the lung tissue of the model group was significantly increased, and the protein expression of GPX4 was significantly decreased. After ATS intervention, the protein expression of Keap1 in the lung tissue of ALI mice was significantly down-regulated, and the protein expression of Nrf2, HO-1, and GPX4 was significantly up-regulated, which was consistent with the effect of the Fer-1 group. These results showed that ATS attenuated LPS-induced ALI via modulation of the Keap1/Nrf2/HO-1 pathway (Fig. 7).

## Discussion

Patients with severe COVID-19 often develop secondary ALI with high morbidity and mortality and lack effective treatment drugs. The late stage typically leads to pulmonary fibrosis and poor prognosis, and seriously reduces the quality of life of patients. Identifying an effective drug with a well-defined mechanism of action for ALI treatment remains an urgent unmet need. The preventive effect of ATS against alveolar cell injury may be related to anti-inflammatory effects and inhibition of oxidative stress. Moreover, it may be involved in regulating the release of EMT factors by promoting the Keap1/Nrf2/HO-1 pathway, which inhibits LPS-induced iron metamorphosis in lung cells.

In ALI, macrophages and neutrophils are activated, numerous cytokines are released, several free radicals are generated, and severe inflammation occurs. ALI leads to destruction of the alveolar epithelial barrier [16]. The severity of lung injury depends on the interaction between inflammatory and alveolar epithelial cells, and the degree of epithelial cell injury. Therefore, in this study, LPS was used to induce type II alveolar cells (A549) to simulate damage to alveolar epithelial cells during ALI [17]. LPS acts as an agonist of the classical inflammatory pathway and can induce the release of inflammatory factors, including TNF-α, IL-6, and IL-1β [18]. ATS significantly inhibits the mRNA expression levels of TNF-α, IL-6, and IL-1β in A549 cells during inflammatory injury; moreover, it inhibits the release of ROS in injured cells thereby reducing the inflammatory injury of alveolar epithelial cells.

Alveolar epithelial cells produce cytokines and chemokines after inflammatory injury, and the continuous generation of ROS is considered a key factor in inducing inflammatory injury [19]. ROS activates the NF-κB dimer to promote its transfer to the nucleus and its transcription. This process leads to an increased expression of proinflammatory cytokines [20]. Excessive and continuous expression of inflammatory factors can also induce pathological fibrosis, and IL-1β can activate EMT-related pathways [21]. TNF-α plays a role through various signalling pathways [22] and regulates alveolar apoptosis and fibrosis. According to previous reports [23], NF-κB is a key central mediator in the EMT process. The activation of NF-κB in cells can inhibit the expression of E-cadherin and down-regulate its expression, which is a key initiation event in EMT [24], thereby promoting lung tissue fibrosis. ATS can inhibit the activation of NF-κB, resulting in increased expression of E-cadherin. ATS also inhibited the increased expression of the fibrosis marker factors α-SMA and MUC5AC.

An important reason for the exacerbation of ALI is the unbalanced inflammatory response in lung lesions, which can promote excessive production of ROS [25]. Oxidative stress occurs when environmental and chemical stimuli increase ROS levels, and the endogenous antioxidant system is unable to repair them [26]. Nrf2 plays a key role in the regulation of intracellular antioxidant molecules and is involved in limiting the gene expression of inflammatory factors. Under physiological conditions, Keap1 forms a complex with Nrf2 and negatively regulates Nrf2. When stress occurs, Keap1, as a redox damage sensor, will receive signals from excess produced oxides, and then Nrf2 will be released from the complex and translocated to the nucleus, gathering in the nucleus [14]. When ALI occurs, the body mobilizes its own antioxidant mechanism, Keap1 and Nrf2 are depolymerized, which is reflected in the increase of Nrf2 expression, and OH-1 is positively regulated by Nrf2, so it is consistent with the trend of Nrf2 expression. However, the antioxidant effect of the body under pathological conditions is not enough to balance oxidative stress. ATS fully mobilizes the activation of Nrf2, which in turn increases the expression of OH-1, and ultimately plays a preventive role in ALI.

GPX4 is a phospholipid hydroperoxide enzyme, which has the ability to inhibit lipid peroxidation and prevent the accumulation of peroxides. It is essential for inhibiting ferroptosis. Inhibition of GPX4 will induce lipid peroxidation. The activity of GPX4 is dependent on glutathione (GSH). When the content of GSH decreases, the activity of GPX4 will be inhibited, and the accumulation of lipid peroxide will occur, which will induce ferroptosis [27]. GSH is an important antioxidant in the human body [28]. As an important cofactor of GPX4, it depends on the regulation of GPX4 to catalyse the reduction of lipid peroxide [29] and further regulate the production of ROS. GSH depletion is considered a sign of ferroptosis [30]. Ferroptosis, an iron-dependent lipid peroxidation-triggered programmed cell death [31], occurs during ALI. Its key features include the generation of massive cellular ROS and dysregulated lipid peroxidation [32]. GPX4 was the first described inhibitor of the ferroptosis centre, and its activity is positively regulated by Nrf2 [33]. Under normal physiological conditions, GSH is at a balanced level. When ALI occurs, the body’s antioxidant balance is out of balance, GSH content decreases, and GPX activity is inhibited. ATS fully mobilizes the activation of Nrf2, positively regulates GPX4, and inhibits the occurrence of ferroptosis.

MPO activity is typically used as an indicator of neutrophil activation and infiltration. Excessively activated MPO can mediate the release of many oxidants, which is closely related to acute lung injury. ATS significantly inhibited the high expression of Keap1 in the lung tissue of ALI mice. Although ATS increased the expression of Nrf2 and HO-1, ATS treatment of ALI was achieved by regulating Keap1/Nrf2/HO-1. ATS decreased the MPO activity in the lung tissue of ALI mice, indicating that it improved the infiltration of inflammatory cells into the lung tissue when ALI occurred. SOD and CAT act as superoxide radicals in the Keap1/Nrf2/HO-1 signalling pathway and can prevent cell damage caused by oxidative stress. ATS significantly up-regulated the expression of GPX4 in the lung tissue of ALI mice and inhibited Fe^2+^ production. Concurrently, the activities of the antioxidant GSH and the superoxide free radicals SOD and CAT in lung tissue were also up-regulated by ATS. ATS plays a role in ALI treatment by inhibiting ferroptosis in lung tissue cells and enhancing the body’s antioxidant capacity.

Oxidative stress and inflammatory responses are two major factors involved in the pathogenesis of ALI. Oxidative stress amplifies the expression of pro-inflammatory genes, which in turn induces ROS production [34]. Translation in Biomedical Scientific Paper Format. In the present study, in vitro cellular experiments were initially conducted to investigate the preventative effects of ATS on the inflammatory response elicited during lung epithelial cell damage, thereby preliminarily verifying the potential of ATS in preventing acute lung injury (ALI). Based on the results of the initial cellular experiments, the potential of ATS in preventing ALI was preliminarily confirmed. Consequently, in vivo animal experiments were utilized to further explore the mechanism by which ATS prevents ALI from the perspective of ferroptosis. The results revealed that ATS inhibited the release of ROS and the mRNA expression levels of TNF-α, IL-6, and IL-1β in damaged lung epithelial cells and played an anti-inflammatory role. It simultaneously inhibits the activation of NF-κB, increases the expression of E-cadherin, and inhibits the expression of α-SMA and MUC5AC to inhibit the fibrosis of lung epithelial cells. ATS exerts antioxidant and anti-inflammatory effects by regulating the Keap1/Nrf2/HO-1/GPX4 pathway and inhibiting tissue Fe^2+^ accumulation.

## Conclusion

Taken together, our results demonstrated that ATS reduced inflammatory injury in A549 cells, suppressed the release of ROS, and inhibited the EMT process induced by sustained cell injury. ATS regulates the signal transduction of the Keap1/Nrf2/HO-1/GPX4 pathway and inhibited LPS-induced ferroptosis-related protein expression and oxidative stress. The present study provides a deeper understanding of the pharmacological efficacy of ATS, which may lead to further successful utilisation of ATS in the clinical treatment of acute lung injury.

## Data Availability

The datasets used and/or analyzed among the current study are available from the corresponding author on reasonable request.

## Abbreviations

ATS: *Aconitum tanguticum* (Maxim.) Stapf
COVID-19: Cronavirus disease
ALI: Acute lung injury
AKT1: Protein kinase B
ALI: Acute lung injury
Bcl-2: B cell lymphoma-2
CAT: Catalase
CMC-Na: Carboxymethylcellulose sodium
DHE: Dihydroethidium
DMSO: Dimethyl sulfoxide
EGFR: Epidermal growth factor receptor
EMT: Epithelial-mesenchymal transition
Fer-1: Ferrostatin-1
GPX4: Glutathione peroxidase 4
GSH: Glutathione
H&E: Haematoxylin & eosin
HO-1: Haeme oxygenase-1
LC-MS/MS: Liquid chromatography-tandem mass spectrometry
IL-1β: Interleukin-1β
IL-8: Interleukin-8
iNOS: Inducible nitric oxide synthase
JAK2: Janus kinase 2
Keap1: Kelch-like ECH-associated protein 1
LPS: Lipopolysaccharides
MMPs: Matrix metalloproteinases
MPO: Myeloperoxidase
MTT: 3-(4,5-Dimethyl-2-thiazolyl)-2,5-diphenyl-2-H-Tetrazolium bromide
NF-κB: Nuclear factor-kappa B
Nrf2: Nuclear factor erythroid 2
PBS: Phosphate buffer saline
QRT-PCR: Quantitative real-time PCR
ROS: Reactive oxygen species
SPF: Specific pathogen-free
SOD: Superoxide dismutase
TNF-α: Tumour necrosis factor-α
TUNEL: Terminal deoxynucleotidyl transferase dUTP nick end-labelling
α-SMA: α-Smooth muscle actin
